# Hypertonic sodium lactate improves fluid balance and hemodynamics in porcine endotoxic shock

**DOI:** 10.1186/s13054-014-0467-3

**Published:** 2014-08-14

**Authors:** Thibault Duburcq, Raphaël Favory, Daniel Mathieu, Thomas Hubert, Jacques Mangalaboyi, Valery Gmyr, Laurence Quintane, Patrice Maboudou, François Pattou, Mercé Jourdain

**Affiliations:** Inserm U859, F-59000 Lille, France; European Genomic Institute for Diabetes (EGID), FR 3508, F-59000 Lille, France; UNIV LILLE 2, F-59000 Lille, France; Pole de Réanimation CHRU, F-59000 Lille, France; Centre de Biologie Pathologie CHRU, F-59000 Lille, France

## Abstract

**Introduction:**

Based on the potential interest in sodium lactate as an energy substrate and resuscitative fluid, we investigated the effects of hypertonic sodium lactate in a porcine endotoxic shock.

**Methods:**

Fifteen anesthetized, mechanically ventilated pigs were challenged with intravenous infusion of E. coli endotoxin. Three groups of five animals were randomly assigned to receive 5 mL/kg/h of different fluids: a treatment group received hypertonic sodium lactate 11.2% (HSL group); an isotonic control group receiving 0.9% NaCl (NC group); a hypertonic control group with the same amount of osmoles and sodium than HSL group receiving hypertonic sodium bicarbonate 8.4% (HSB group). Hemodynamic and oxygenation variables, urine output and fluid balance were measured at baseline and at 30, 60, 120, 210 and 300 min. Skin microvascular blood flow at rest and during reactive hyperemia was obtained using a laser Doppler flowmetry technique. Results were given as median with interquartile ranges.

**Results:**

Endotoxin infusion resulted in hypodynamic shock. At 300 min, hemodynamics and oxygenation were significantly enhanced in HSL group: mean arterial pressure (103 [81–120] mmHg vs. 49 [41–62] in NC group vs. 71 [60–78] in HSB group), cardiac index (1.6 [1.2–1.8] L/min/m^2^ vs. 0.9 [0.5–1.1] in NC group vs. 1.3 [0.9–1.6] in HSB group) and partial pressure of oxygen (366 [308–392] mmHg vs. 166 [130–206] in NC group vs. 277 [189–303] in HSB group). At the same time, microvascular reactivity was significantly better in HSL group with a lower venoarterial CO_2_ tension difference (5.5 [4–10] mmHg vs. 17 [14–25] in NC group vs. 14 [12–15] in HSB group). The cumulative fluid balance was lower in HSL group (-325 [-655; -150] mL) compared to NC (+560 [+230; +900] mL, p = 0.008) and HSB (+185 [-110; +645] mL, p = 0.03) groups.

**Conclusions:**

In our hypodynamic model of endotoxic shock, infusion of hypertonic sodium lactate improves hemodynamic and microvascular reactivity with a negative fluid balance and a better oxygenation.

**Electronic supplementary material:**

The online version of this article (doi:10.1186/s13054-014-0467-3) contains supplementary material, which is available to authorized users.

## Introduction

Sepsis, the syndrome of microbial infection complicated by systemic inflammation, is a major public health problem associated with significant morbidity and mortality [1,2]. Due to constant hypovolemia, fluid resuscitation is a cornerstone of septic shock management [3]. Indeed, early fluid therapy is common in patients with septic shock [4]. Both adequate initial fluid resuscitation and conservative late fluid management could improve patient outcome [5]. In fact, excess fluid could be deleterious [6], and negative fluid balance has been shown to be associated with better survival rates [7], especially in acute lung injury [8,9], and acute kidney injury [10]. Although adequate fluid resuscitation is commonly acknowledged as an important element in the treatment of patients with septic shock, the choice of resuscitation fluid remains a point of discussion. The 2012 Sepsis Surviving Campaign guidelines recommend crystalloids as the initial fluid of choice for resuscitation in severe sepsis and septic shock [11]. Isotonic saline (0.9% sodium chloride) is the most commonly used fluid [12]. Nevertheless, in order to avoid excess fluid, the concept of small volume resuscitation with hypertonic saline solute has been well-received. Low volume of hypertonic fluids may have valuable effects on restoration of intravascular volume (fluid shifts from the intracellular to the extracellular compartment), on improvement of cardiac output and vascular tone [13], and on improvement of regional microcirculation without fluid overload [14]. However, with the potentially beneficial sodium cation, the presence of non-metabolized chloride anion may have detrimental effects. Supraphysiological concentrations of chloride, which induce hyperchloremia and metabolic acidosis, may cause renal vasoconstriction and decreased glomerular filtration rate [15]. Moreover, in a recent study, a chloride-restrictive strategy in intensive care units was associated with a significant decrease in the incidence of acute kidney injury and in the use of renal replacement therapy [16]. Hence, in an attempt to avoid the detrimental effects of non-metabolized anion, the use of metabolized anions such as lactate may be required. The use of lactate is an interesting alternative because this anion is well-metabolized [17] even in poor hemodynamic conditions [18]. Some positive clinical experience in using sodium lactate is available. In cardiac surgery, exogenous lactate has been shown to improve cardiac index and has been shown to be safe and well-tolerated [19]. Lactate may serve as a resuscitation fluid-based energetic substrate providing a high-octane fuel [20] to improve heart performance [19,21], and simultaneously to normalize fluid balance [14,21]. There are no data available on the administration of hypertonic sodium lactate in sepsis or septic shock. Based on the potential interest in sodium-lactate as an energy substrate and resuscitative fluid, we investigated the effects of hypertonic sodium lactate on fluid balance, hemodynamic and microcirculation in a porcine model of endotoxic shock.

## Materials and methods

We performed a prospective, randomized, controlled experimental study approved by the Institutional Review Board for Animal Research (protocol CEEA 132012); care and handling of the animals were in accordance with National Institutes of Health guidelines.

### Animal preparation

Fifteen adult female Large White pigs (2 months old) were used in this study. For the experiment, animals were premedicated with intramuscular injection of ketamine (Kétalar®, Virbac, Vauvert, France, 2.5 mg/kg of body weight) and xylazine (Sédaxylan®, CEVA Santé Animale, Libourne, France, 2.5 mg/kg of body weight). Then we used Isoflurane (Aerrane®, Baxter, Maurepas, France) for the intubation process, and maintenance of anesthesia was performed with a continuous infusion of midazolam (Hypnovel®, Roche, Neuilly, France, 1 to 2 mg/kg body weight/h) for the whole experiment. Animals were mechanically ventilated (Osiris 2®, Taema, Antony, France) with a tidal volume 10 mL/kg, positive end-expiratory pressure 3 cmH_2_O to limit cardiovascular effects, inspired oxygen fraction (FiO_2_) 0.6 to prevent fatal hypoxemia during the study, respiratory rate 14 to 20 breaths/minute only adjusted to have normocapnia (40 to 45 torr) at baseline. We chose to maintain similar ventilation in all animals during the experiment. No recruitment maneuvers were done. Muscle relaxation was obtained by a continuous infusion of cisatracurium besylate (Nimbex®, Hospira, Meudon, France, 2 mg/kg body weight/h). Analgesia was achieved by a subcutaneous injection of buprenorphine (Vetergesic®, Sogeval, Laval, France, 0.1 mg/kg body weight). Subcutaneous administration of buprenorphine in our animals is known to permit analgesia during six to eight hours. After dissection of neck vessels, catheters were inserted in the pulmonary artery via the right external jugular vein (Swan-Ganz; Baxter 110 H 7.5 F; Baxter Edwards Critical Care, Irvine, CA, USA) and in the right carotid artery for continuous blood pressure monitoring and blood sampling. To monitor urine output, a suprapubic urinary catheter was inserted by laparotomy. An esophageal temperature probe measured core temperature.

### Macrocirculatory and oxygenation parameters

Systemic arterial and venous blood samples from carotid and pulmonary arteries were obtained simultaneously. Arterial and venous blood gas tensions and lactate levels were measured in an acid-base analyzer (ABL-800, Radiometer, Copenhagen, Denmark). Blood oxygen content was calculated from the hemoglobin content and oxygen saturation. Heart rate, systemic and pulmonary arterial pressures were continuously monitored (90308 PC Express Portable Monitor, Spacelabs, Snoqualmie, WA, USA) as well as cardiac output and mixed venous oxygen saturation (Vigilance monitor; Baxter Edwards, Irvine, CA, USA). Using standard formulae we computed global oxygen delivery (DO_2_), global oxygen consumption (VO_2_), oxygen extraction ratio (OER), cardiac index (L/min/m^2^), cardiac power index (CPI) (W/m^2^) [22], systemic vascular resistance (SVR) and pulmonary vascular resistance (PVR) (dynes/sec/cm^5^). Body surface area was calculated by Kelley’s formula [23].

### Study design

**Figure 1:**
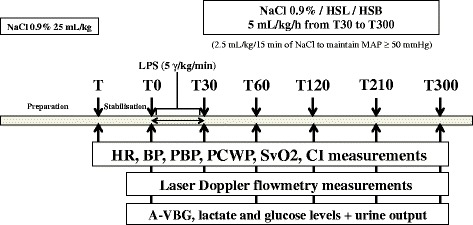
**Study design.** During the preparation period, animals received 25 mL/kg 0.9% NaCl to prevent hypovolemia. When preparation was completed, a 30-minute period served to stabilize the measured variables. Measurements were taken over a 5-h period. Throughout the duration of the experiment, heart rate (HR), blood pressure (BP), pulmonary blood pressure (PBP), pulmonary capillary wedge pressure (PCWP), mixed venous oxygen saturation (SvO2), cardiac index (CI) and laser Doppler flowmetry measurements were recorded. Arterial and venous blood gas (A-VBG) was collected at the same time (except T and T30) until the study was completed. Animals were infused with 5 μg/kg/min of *Escherichia coli* lipopolysaccharide (LPS) from T0 to T30. We studied three groups of five animals receiving 5 mL/kg/h of different fluids: 0.9% NaCl (NC group), hypertonic sodium bicarbonate 8.4% (HSB group) and hypertonic sodium lactate 11.2% (HSL group). If mean arterial pressure (MAP) felt below 50 mmHg, a 2,5 mL/kg infusion of NaCl 0.9% was given as rescue therapy every 15 minutes. Bolus infusions were performed to maintain mean arterial pressure (MAP) above 50 mmHg.

The study was carried out as depicted in Figure 1. During the preparation period, animals received 25 mL/kg 0.9% NaCl to prevent hypovolemia. When all preparations were completed, a 30-minute period was allowed to stabilize the measured variables. Macrocirculatory measurements were taken over a 5-h period: two times at baseline (before (T) and after (T0) the stabilization period) and at 30 (T30), 60 (T60), 120 (T120), 210 (T210) and 300 (T300) minutes. Arterial and venous blood gas tensions and lactate levels were collected at the same time (except T and T30) until the study was completed. After anesthesia, catheterization, and baseline collection (T0), all animals were administered 5 μg/kg/min *Escherichia coli* lipopolysaccharide (LPS) (serotype 055:B5; Sigma Chemical Co., St. Louis, MO, USA). The endotoxin was diluted in 50 mL of 0.9% NaCl and infused intravenously over a 30-minute period. Our model was of hypodynamic shock with low level resuscitation. The only resuscitation endpoint was mean arterial pressure (MAP). We studied three groups of five animals receiving 5 mL/kg/h (from T30 to T300) of different fluids: two control groups, receiving 0.9% NaCl (NC group), 8.4% hypertonic sodium bicarbonate (HSB group) containing 61 g of bicarbonate and 23 g of sodium per liter and a treatment group receiving 11.2% hypertonic sodium lactate AP-HP® (AGEPS, Paris, France) (HSL group) containing 90 g of lactate and 23 g of sodium per liter (Additional file 1). Hypertonic groups (HSB and HSL groups) provided the same amount of sodium and osmoles (2,000 mosm/L). If MAP fell below 50 mmHg, a 2.5-mL/kg infusion of NaCl 0.9% was given as rescue therapy every 15 minutes. Bolus infusions were performed to maintain MAP above 50 mmHg.

### Microcirculatory parameters

Skin microvascular blood flow was measured continuously using a laser Doppler flowmeter probe and device (Periflux® PF407; Perimed, Jiirfalla, Sweden). The blood flow was measured in a volume of 1 mm^3^ solid tissue. The fiber optic probe was applied on the right hind paw of the animals and fixed with adhesive tape. Laser Doppler signal was continuously registered on a personal computer. Readjusting the pen of the recorder to zero when the probe was fixed to a white non-moving surface performed flux zero calibration. Skin blood flow was measured at rest and during reactive hyperemia from T0 to T300, and values were expressed in arbitrary perfusion units (PU). Reactive hyperemia was produced by arrest of leg blood flow with a pneumatic cuff inflated to a suprasystolic pressure (200 mmHg) for 3 minutes. On completion of the ischemic period, the occluding cuff was rapidly deflated to zero. Peak flow was defined as the highest flow signal during the post occlusive phase. Reactive hyperemia was further analyzed (Perisoft® 2.5 software) according to its duration and initial reactive hyperemia uphill slope.

### Biological methods and fluid balance

During the study, capillary glucose (Accu-Chek PERFORMA®, Roche Diagnostics SAS, Meylan, France) was measured at T0, T60, T120, T210 and T300. Other variables included arterial serum and urinary electrolyte concentrations, urea, creatinine and measured osmolalities. Apparent strong ion difference (SID) was calculated as:

((Na^+^ + K^+^ + Ca^2+^ + Mg^2+^) – (Cl^-^ + Lactate^-^)) [24].

Variables were recorded at T0 and T300. At the end of the study, sodium and chloride balance was determined by calculating for each individual the difference between the cumulative amounts of electrolytes administered from T210 to T300 and the cumulative amounts of electrolytes collected in the urine during the same period. We also computed urea and creatinine clearance with standard formulas. Urine output was measured at T0, T60, T120, T210 and T300. Fluid balance from T0 to T300 was determined by calculating the difference between the cumulative amounts of fluids administered and the cumulative amounts of fluids collected in the urine.

### Data analysis

Statistical analysis was performed with GraphPad Prism 6 software. As the distribution was not normal (Shapiro-Wilk test), quantitative data were expressed using median and interquartile range. For multiple intergroup testing, we used the Kruskal-Wallis test with the Dunn multiple comparisons test and the Mann-Whitney *U*-test. Intragroup comparisons were performed using the Friedman test with the Dunn multiple comparisons test. A *P*-value <0.05 was considered significant.

## Results

The fifteen studied animals were divided into three groups. Median weight was similar in the three groups of animals: 21 (19.25 to 22.75) kg in the NC group, 21 (19.75 to 22.5) kg in the HSB group and 22 (20.75 to 22.5) kg in the HSL group.

### Evolution of lactate levels

Lactate levels (Additional file 2) were comparable at baseline between the three groups (0.7 (0.7 to 0.8) mmol/L). During the study, we observed a similar increase of lactate levels in both control groups. At 300 minutes, lactate levels were 5.5 (3.8 to 8) mmol/L in the NC group and 4.2 (3.2 to 7.9) mmol/L in the HSB group. In the HSL group, however, lactate levels increased earlier. At 60 minutes, lactate level was 9.7 (9.3 to 14) mmol/L and then remained stable until the end of the study. Compared with control groups, lactate levels were significantly higher in the HSL group from T60 to T300 (*P* <0.0001).

### Macrocirculatory parameters

**Figure 2:**
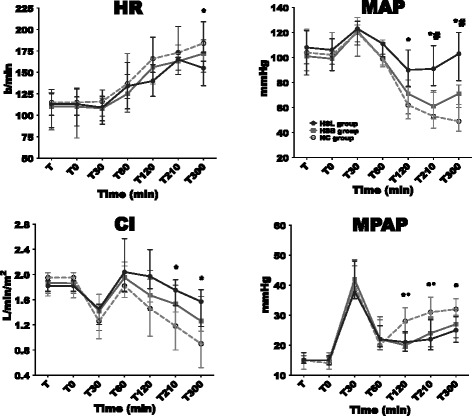
**Changes in heart rate (HR), mean arterial pressure (MAP), cardiac index (CI) and mean pulmonary arterial pressure (MPAP).** Open circles and dotted line: 0.9% NaCl (NC) group (n = 5); squares and gray line: hypertonic sodium bicarbonate (HSB) group (n = 5); closed circles and black line: hypertonic sodium lactate (HSL) group (n = 5). Results are expressed as median with interquartile range. When not displayed, error bars are within the symbols. *P <0.05, NC versus HSL; ^#^ *P* <0.05, HSB versus HSL; °*P* <0.05, NC versus HSB.

**Figure 3:**
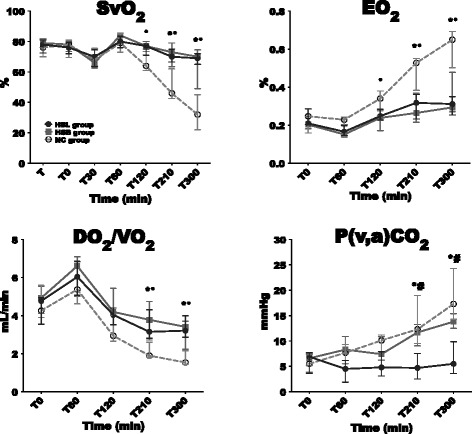
**Changes in mixed venous oxygen saturation (SvO**_**2**_ **), oxygen extraction (EO**_**2**_ **), DO**_**2**_ **/VO**_**2**_ **ratio and venoarterial CO2 tension difference (Pv-aCO**_**2**_ **).** Open circles and dotted line: 0.9% NaCl (NC) group (n = 5); squares and gray line: hypertonic sodium bicarbonate (HSB) group (n = 5); closed circles and black line: hypertonic sodium lactate (HSL) group (n = 5). Results are expressed as median with interquartile range. When not displayed, error bars are within the symbols. *P <0.05, NC versus HSL; ^#^ *P* <0.05, HSB versus HSL; °*P* <0.05, NC versus HSB.

Changes in HR, MAP, CI, mean pulmonary arterial pressure (MPAP), mixed venous oxygen saturation (SvO_2_), oxygen extraction (EO)_2_, DO_2_/VO_2_ ratio and Pv-aCO_2_ are illustrated in Figures 2 and 3. Variations in right atrial pressure (RAP), pulmonary capillary wedge pressure (PCWP) and CPI are presented in Additional file 2. No changes in studied variables were observed during stabilization period and no differences were observed among groups. Endotoxin infusion resulted in similar and usual changes in hemodynamics until 60 minutes as previously described [25,26]. Thereafter, better hemodynamic stability was observed in the HSL group. At 300 minutes, MAP returned to the baseline level in the HSL group while a 51% and 30% decrease was observed respectively in the NC and HSB groups. Throughout the study, six animals (three in the NC group, two in the HSB group and only one in the HSL group) required 0.9% NaCl boluses to maintain MAP ≥50 mmHg according to the study protocol. SVR changes were similar in the three groups without any significant differences. CI impairment, at 300 minutes, was less pronounced in the HSL group (1.6 (1.2 to 1.8) L/min/m^2^) compared to BS (1.3 (0.9 to 1.6), *P* = 0.53) and NC (0.9 (0.5 to 1.1), *P* = 0.01) groups. Finally, compared to control groups, CPI was significantly better from 120 to 300 minutes in the HSL group. With regard to lung function, we observed a similar increase (60% compared to baseline) in MPAP 30 minutes after the infusion of endotoxin in the three groups, then MPAP decreased at 60 minutes. From 120 to 210 minutes, the increase in MPAP was significantly more important in the NC group compared with the hypertonic groups. DO_2_ decreased in the three groups between 60 and 300 minutes. Compared with hypertonic groups, this decrease was significantly more pronounced in the NC group from 210 to 300 minutes. At the same time, VO_2_ increased in the three groups without significant differences, resulting in increased EO_2_ in the three groups. Compared to the hypertonic groups, this EO_2_ increase was significantly more important in the NC group from 210 to 300 minutes. In parallel, SvO_2_ decreased from baseline to 300 minutes by 56% in the NC group, 18% in the HSB group and 9% in the HSL group. They were no significant differences between the hypertonic groups in DO_2_, EO_2_ and SvO_2_ changes. Nevertheless, venoarterial CO2 tension difference (P(v-a)CO_2_) was larger in the HSB group (14 (12 to 15)) than in the HSL group (5.5 (4 to 10), *P* = 0.01).

### Microcirculatory parameters

**Figure 4:**
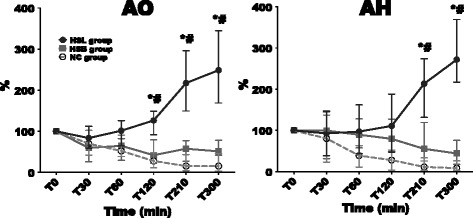
**Variations in occlusive areas (AO) and hyperaemic areas (AH).** Open circles and dotted line: 0.9% NaCl (NC) group (n = 5); squares and gray line: hypertonic sodium bicarbonate (HSB) group (n = 5); closed circles and black line: hypertonic sodium lactate (HSL) group (n = 5). Results are expressed as median with interquartile range. When not displayed, error bars are within the symbols. *P <0.05, NC versus HSL; ^#^ *P* <0.05, HSB versus HSL; °*P* <0.05, NC versus HSB. PU, perfusion units.

Changes in occlusive areas (AO) and hyperemic areas (AH) are illustrated in Figure 4. Occlusive areas (AO), hyperemic areas (AH), rest flow (RF) and peak flow (PF) were similar at baseline between the three groups. During the study period, all these variables decreased significantly in the NC group and increased significantly in the HSL group. No significant variations were observed in the HSB group. At the end of the study (300 minutes), RF and PF were better in the HSL group (33 (22 to 40) PU and 58 (50 to 84) PU respectively) compared to the NC (8 (7 to 11) PU; *P* = 0.01 and 15 (14 to 17) PU; *P* = 0.01 respectively) and HSB groups (18 (13 to 23) PU; *P* = 0.03 and 37 (30 to 39) PU; *P* = 0.01 respectively). In the same way, AO and AH at 300 minutes were more important in the HSL group (3,200 (2,300 to 6,800) PU × sec and 2,300 (700 to 3,500) PU × sec respectively) compared to the NC (270 (120 to 370) PU × sec; *P* = 0.01 and 110 (31 to 200) PU × sec; *P* = 0.01 respectively) and HSB groups (600 (200 to 1,300) PU × sec; *P* = 0.04 and 800 (300 to 1,400) PU × sec; *P* = 0.15 respectively).

### Urine output and fluid balance

**Figure 5:**
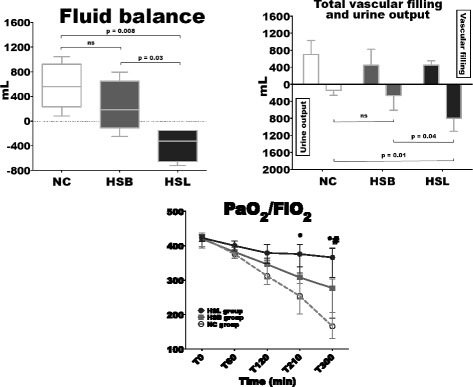
**Total vascular filling, urine output, fluid balance from time zero (T0) to time 300 minutes (T300) and variations in arterial oxygen partial pressure and inspired oxygen fraction ratio (PaO**_**2**_ **/FiO**_**2**_ **).** Open circles and dotted line: 0.9% NaCl (NC) group (n = 5); squares and gray line: hypertonic sodium bicarbonate (HSB) group (n = 5); closed circles and black line: hypertonic sodium lactate (HSL) group (n = 5). Results are expressed as median with interquartile range. When not displayed, error bars are within the symbols. *P <0.05, NC versus HSL; ^#^ *P* <0.05, HSB versus HSL; °*P* <0.05, NC versus HSB.

Total vascular filling, urine output, fluid balance and the variations in arterial oxygen partial pressure and inspired oxygen fraction (PaO_2_/FiO_2_) ratio are illustrated in Figure 5. Urine output and fluid balance at baseline were similar among groups. From 120 to 300 minutes, significantly higher urine output was noticed in the HSL group compared to control groups, resulting in a significantly lower fluid balance. PaO_2_/FiO_2_ ratio decreased in the three groups. Compared to control groups, this alteration was significantly less pronounced in the HSL group. Median values of PaO_2_/FiO_2_ at 300 minutes were 366 (308 to 392) mmHg in the HSL group versus 277 (189 to 303) (*P* = 0.03) in HBS group and 166 (130 to 206) (*P* = 0.01) in the NC group.

### Biological parameters

The evolution of biological parameters during the study is presented in Table 1. In the NC group, urine tests were not performed at 300 minutes because all animals were anuric. Biological parameters were comparable at T0 between the three groups.

**Table 1:** **Evolution of biological parameters in the three groups**

	**Time (T), minutes**	**0.9% NaCl (NC) group**	**Hypertonic sodium bicarbonate (HSB) group**	**Hypertonic sodium lactate (HSL) group**	***P*** **-value**		
					**NC versus**	**NC versus**	**HSB versus**
					**HSB**	**HSL**	**HSL**
**BLOOD**							
**Lactate, mmol/L**	T0	**0.7 (0.5 to 0.8)**	**0.65 ( 0.6 to 0.7)**	**0.7 (0.7 to 0.8)**	**ns**	**ns**	**ns**
	T210	**3.9 (2.7 to 6.9)**	**3.7 (2.8 to 4.7)**	**11.8 (10.5 to 17.7)**	**ns**	**0.01**	**0.01**
	T300	**5.5 (3.8 to 8.0)**	**4.2 (3.2 to 7.9)**	**11.8 (10.3 to 16.0)**	**ns**	**0.01**	**0.01**
**pH**	T0	**7.45 (7.39 to 7.48)**	**7.44 (7.38 to 7.47)**	**7.43 (7.41 to 7.48)**	**ns**	**ns**	**ns**
	T210	**7.23 (7.21 to 7.26)**	**7.45 (7.37 to 7.50)**	**7.50 (7.46 to 7.53)**	**0.01**	**0.01**	**ns**
	T300	**7.20 (7.17 to 7.24)**	**7.47 (7.42 to 7.52)**	**7.52 (7.47 to 7.55)**	**0.01**	**0.01**	**ns**
**HCO3** ^**-**^ **, mmol/L**	T0	**29 ( 28 to 32)**	**29 (28 to 31)**	**30 (28 to 31)**	**ns**	**ns**	**ns**
	T210	**22 (20 to 26)**	**48 (44 to 52)**	**49 (46 to 54)**	**0.01**	**0.01**	**ns**
	T300	**19 (15 to 25)**	**54 (48 to 59)**	**55 (51 to 58)**	**0.01**	**0.01**	**ns**
**PaCO2, mmHg**	T0	**43 (41 to 47)**	**44 (40 to 49)**	**45 (41 to 48)**	**ns**	**ns**	**ns**
	T210	**50 (44 to 53)**	**65 (49 to 67)**	**58 (49 to 63)**	**ns**	**ns**	**ns**
	T300	**52 (47 to 59)**	**70 (62 to 73)**	**62 (57 to 68)**	**ns**	**ns**	**ns**
**Urea, g/L**	T0	**0.18 (0.13 to 0.21)**	**0.16 (0.13 to 0.19)**	**0.14 (0.11 to 0.20)**	**ns**	**ns**	**ns**
	T300	**0.34 (0.28 to 0.42)**	**0.30 (0.26 to 0.34)**	**0.18 (0.13 to 0.23)**	**ns**	**0.02**	**0.02**
**Creatinine, mg/L**	T0	**9.0 (7.5 to 10.0)**	**7.0 (6.0 to 8.5)**	**7.0 (6.5 to 8.5)**	**ns**	**ns**	**ns**
	T300	**16.0 (13.5 to 17.5)**	**10.0 (9.5 to 14.5)**	**11.0 (10.0 to 12.0)**	**0.04**	**0.02**	**ns**
**Na** ^**+**^ **, mmol/L**	T0	**140 (137 to 145)**	**142 (138 to 146)**	**137 (138 to 140)**	**ns**	**ns**	**ns**
	T300	**142 (138 to 146)**	**164 (161 to 166)**	**162 (158 to 164)**	**0.01**	**0.01**	**ns**
**K** ^**+**^ **, mmol/L**	T0	**4.0 (3.9 to 4.1)**	**3.9 (3.5 to 4.3)**	**3.9 (3.7 to 4.0)**	**ns**	**ns**	**ns**
	T300	**4.9 ( 4.6 to 5.4)**	**3.7 (3.5 to 3.9)**	**3.5 (3.3 to 3.6)**	**0.01**	**0.01**	**ns**
**Cl** ^**-**^ **, mmol/L**	T0	**101 (98 to 107)**	**101 (100 to 105)**	**100 (99 to 101)**	**ns**	**ns**	**ns**
	T300	**106 (103 to 115)**	**98 (96 to 101)**	**93 (91 to 96)**	**0.02**	**0.01**	**0.02**
**Ca2** ^**+**^ **, mmol/L**	T0	**2.48 (2.37 to 2.51)**	**2.55 (2.31 to 2.59)**	**2.45 (2.37 to 2.57)**	**ns**	**ns**	**ns**
	T300	**2.1 (1.8 to 2.2)**	**1.9 (1.8 to 2)**	**2.0 (1.9 to 2.2)**	**ns**	**ns**	**ns**
**Mg2** ^**+**^ **, mmol/L**	T0	**0.75 (0.70 to 0.80)**	**0.68 (0.64 to 0.73)**	**0.70 (0.68 to 0.77)**	**ns**	**ns**	**ns**
	T300	**1.0 (0.9 to 1.1)**	**0.74 (0.66 to 0.88)**	**0.70 (0.64 to 0.80)**	**0.02**	**0.02**	**ns**
**SID, mEq/L**	T0	**45 (42 to 47)**	**44 (39 to 45)**	**45 (44 to 46)**	**ns**	**ns**	**ns**
	T300	**36 (32 to 41)**	**67 (60 to 70)**	**64 (61 to 68)**	**0.01**	**0.01**	**ns**
**Osmolality, mosm/kg**	T0	**294 (289 to 304)**	**290 (286 to 295)**	**293 (286 to 296)**	**ns**	**ns**	**ns**
	T300	**302 (295 to 308)**	**337 (335 to 342)**	**341 (338 to 345)**	**0.01**	**0.01**	**ns**
**URINE**							
**Na** ^**+**^ **, mmol/L**	T0	**131 (78 to 150)**	**91 (69 to 150)**	**95 (84 to 110)**	**ns**	**ns**	**ns**
	T300		**111 (90 to 156)**	**183 (169 to 190)**			**0.03**
**K** ^**+**^ **, mmol/L**	T0	**22.3 (16.6 to 38.6)**	**15.3 (9.3 to 32.9)**	**23.4 (13.6 to 28.8)**	**ns**	**ns**	**ns**
	T300		**24.6 (13.1 to 36.2)**	**15.2 (10.7 to 17.9)**			**ns**
**Cl** ^**-**^ **, mmol/L**	T0	**157 (64 to 183)**	**125 (64 to 197)**	**98 (83 to 137)**	**ns**	**ns**	**ns**
	T300		**49 (31 to 55)**	**51 (46 to 52)**			**ns**
**Osmolality, mosm/kg**	T0	**543 (309 to 683)**	**475 (339 to 576)**	**394 (252 to 468)**	**ns**	**ns**	**ns**
	T300		**366 (328 to 377)**	**378 (362 to 383)**			**ns**

Results are expressed as median with interquartile range. Mann-Whitney *U*-test was used for intergroup comparisons; *P* <0.05 was considered significant. Urine tests were not performed at 300 minutes in the NC group because all animals were anuric. SID, apparent strong ion difference ((Na^+^ + K^+^ + Ca^2+^ + Mg^2+^) – (Cl^-^ + Lactate^-^)).

#### Acid-base status

Arterial pH and bicarbonate levels decreased over time (*P* <0.01 at 300 minutes) in the NC group. Contrarily, these variables levels increased over time in the hypertonic groups (*P* <0.001 at 300 minutes) without any differences between the HSB and HSL groups. Compared to the NC group, pH and bicarbonate levels were significantly higher in the HSB and HSL groups from 120 to 300 minutes. The partial pressure of carbon dioxide increased in the three groups over time without any significant differences.

#### Electrolyte balance and renal function

Sodium and chloride intake, from 210 to 300 minutes, was comparable in the HSB and HSL groups. However sodium and chloride output was higher in the HSL group (50 (17 to 77) and 13 (5 to 20) mmol respectively) compared to the HSB group (1.1 (0.4 to 12) mmol, *P* = 0.02 and 0.4 (0.3 to 3.2) mmol, *P* = 0.004 respectively), thus resulting in significantly lower sodium and chloride balances in the HSL group. Urea and creatinine clearance was also significantly higher in the HSL group (40 (13 to 52) and 51 (27 to 79) mL/minute) compared to the HSB group (4 (1 to 11) and 12 (3 to 24) mL/minute, *P* = 0.01 and 0.03 respectively).

#### Blood glucose levels

**Figure 6:**
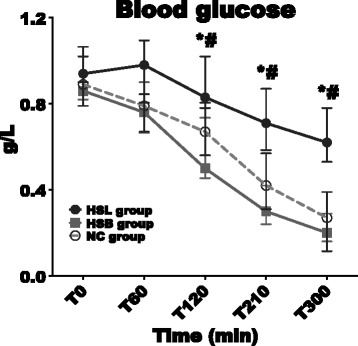
**Variations in blood glucose levels.** Open circles and dotted line: 0.9% NaCl (NC) group (n = 5); squares and gray line: hypertonic sodium bicarbonate (HSB) group (n = 5); closed circles and black line: hypertonic sodium lactate (HSL) group (n = 5). Results are expressed as median with interquartile range. When not displayed, error bars are within the symbols. *P <0.05, NC versus HSL ^#^ *P* <0.05; HSB versus HSL; °*P* <0.05, NC versus HSB.

We observed a significant decrease in both control groups while it remained stable in the HSL group (Figure 6). At 300 minutes, blood glucose levels were significantly higher in the HSL group (0.62 (0.53 to 0.78) g/L) compared to the NC (0.27 (0.12 to 0.39) g/L, *P* = 0.01) and HSB (0.20 (0.16 to 0.29) g/L, *P* = 0.01) groups.

## Discussion

We report here that the infusion of HSL solution improves hemodynamic stability and microvascular reactivity with a negative fluid balance and better oxygenation. This was the first time that HSL was investigated in large endotoxic animals. Pigs were chosen as a clinically relevant species, resembling humans in various functions as assessed by cardiovascular, respiratory, and biochemical parameters [27]. In order to maximize the potential beneficial effects of HSL, we deliberately investigated its impact in the worst hemodynamic conditions associated with sepsis. In fact, our model was that of hypodynamic shock with low-level resuscitation. We used a low rate of vascular filling to maintain MAP ≥50 mm Hg rather than keeping the volume constant and allowing pressures to vary. Our purpose was to compare HSL with two different therapeutic regimens: a standard fluid therapy with isotonic crystalloids (0.9% NaCl, the most commonly used crystalloid) and a non-conventional hypertonic fluid therapy with the same amount of osmoles and sodium. Due to an acidifying effect on pH and an elevated chloride concentration, hypertonic saline was not close enough to HSL. We decided that the best hypertonic control was HSB. In fact, HSB does not contain a chloride cation and has the same alkaline effect on blood pH as HSL (see Table 1). On HSL infusion, lactate is metabolized and the remaining Na^+^ increases the SID, leading in turn to decreased water dissociation and proton concentration, resulting in an alkalinizing effect [24]. Thereby, the main difference between hypertonic solutions was the potential role of lactate as an energetic substrate.

The hemodynamic results of our study were consistent with a favorable effect of hypertonic solutions. An explanation could be the larger amount of sodium, which regulated extracellular volume, in the hypertonic groups. This is in good agreement with data from the literature [28,29]. Potential advantages of hypertonic fluid administration rather than isotonic solutions have already been described in shock [30]. High extracellular sodium concentration could favor a reverse mode of Na^+^-Ca^2+^ exchanger to induce a positive inotropic effect [31]. These hemodynamic improvements were more marked in the HSL compared to the HSB group. MAP in the HSL group was completely normalized at the end of the study and was significantly higher compared to the HSB group. The hemodynamic effects of lactate seemed to be related to improvement in cardiac function rather than changes in vascular tone. In fact, SVR was not significantly modified by lactate administration. Moreover, the CI was slightly higher in the group receiving lactate as has already been observed [19]. It is known that lactate improves cardiac efficiency during shock [32,33], and it has been shown that lactate deprivation during shock impairs heart metabolism [34]. Despite the fact that hypertonic fluids contained the same amounts of sodium, HSB and HSL were different in term of calories. These findings support the role of lactate as a key metabolic substrate under stress conditions [35,36]. Furthermore, P(v-a)CO_2_ gradient was significantly lower and skin blood flow, at rest and during hyperemia, was significantly better in HSL group, suggesting better tissue perfusion. Hypertonic fluids are known to reduce microvascular collapse, restoring vital nutritional blood flow and tending to blunt the upregulation of leukocyte and endothelial adhesion molecules [37]. The improvement of microcirculation in the HSL group compared to the HSB group could be the result of an overall improvement in global perfusion (MAP), and maybe an intrinsic effect of lactate that remains to be explored.

One remarkable result of this study was the concurrent improvement of hemodynamic status and plasma volume expansion in the HSL group, whereas fluid balance was negative. The higher volume of urine output in the HSL group reflected an improvement in renal perfusion that could be explained by better macro- and microcirculatory status. Moreover, we observed a positive sodium balance associated with a negative chloride balance following HSL infusion, which supports chloride urine excretion coupled to the high sodium urine excretion. In fact, the important amount of urinary sodium excretion creates an imbalance between positive and negative charges. As the urine is poor in protein (positive charges could be neutralized by the increase of negative charges on protein), a net anion efflux must therefore compensate the excess of positive charges in order to maintain electroneutrality. This is achieved for a substantial part by an increase of urinary chloride excretion [38,39]. Chloride, the principal intracellular inorganic anion, is responsible for intracellular tonicity; hence the net efflux of chloride could be accompanied by a net flux of water [40]. This could participate to the larger urine output with HSL infusion. This finding is noteworthy because it is generally difficult to achieve a negative fluid balance in the early phase of septic shock. Reducing fluid overload and edema are always regarded as having positive effects because of their link with organ dysfunction [6,10]. In a previous study in the same animal model, histologic findings supported the development of respiratory distress syndrome with marked pulmonary leukocyte sequestration and interstitial edema [26]. In our study, the negative fluid balance in the lactate group was associated with improved PaO_2_/FiO_2_ ratio. This was consistent with a previous human study on conservative strategy of fluid management [8]. Finallyt, as already described [41], the development of hypoglycemia in porcine endotoxic shock could be due to a peak in the insulin level at 90 minutes. Stability in blood glucose levels in the animals receiving HSL was anticipated because lactate is a good precursor for glucose through liver gluconeogenesis.

Our model was stable with no hemodynamic variations during the stabilization period. We observed that HSL resulted in significant improvements despite the small numbers of animals per group. This suggests an important effect of HSL. Hence, two main mechanisms could be advanced to explain this effect: exogenous lactate infusion is beneficial as an energy supplier and the resulting sodium/chloride imbalance may induce a negative fluid balance. However, our model presented some limits: the short duration (five hours only, so that there was little time for activation of the NO system); administration of an LPS bolus rather than continuous intravenous administration (bolus produces much more severe acute pulmonary hypertension and eventually right ventricular failure); and hypodynamic shock with inadequate resuscitation, whereas hyperdynamic shock is more common in the ICU. Moreover, we observed predictable side effects of HSL with hypernatremia and metabolic alkalosis. It should be more suitable to use half molar sodium lactate or to decrease the dose of 11.2% HSL in order to limit the increase in sodium and bicarbonate levels. Furthermore, infusion of such a large amount of lactate will modify the serum lactate level and kinetics, and will have to be taken into account in interpreting lactate monitoring during the initial resuscitation. Last, if HSL was better that HSB due to lactate metabolism, it would be interesting to add another energy substrate (glucose for example) to HSB in future experiments.

## Conclusion

HSL can be used as an energy substrate and resuscitative fluid. In our hypodynamic model of endotoxic shock, HSL infusion improved hemodynamic stability and microvascular reactivity with a negative fluid balance and better oxygenation. Further investigations are warranted to assess the potential clinical benefits of this treatment.

## Key messages

- Lactate is an interesting energetic substrate that is well-metabolized even in poor hemodynamic conditions and hypertonic sodium lactate can be used as a resuscitative fluid.
- In our porcine model of endotoxic shock, infusion of hypertonic sodium lactate improves hemodynamics and microcirculation with a negative fluid balance and better oxygenation.
- The resulting sodium/chloride imbalance with hypertonic sodium lactate may induce a negative fluid balance.

## Additional files

Additional file 1:
**Composition of 0.9% NaCl, 8.4% hypertonic sodium bicarbonate (HSB) and 11.2% hypertonic sodium lactate (HSL).**
Additional file 2:
**Evolution of right atrial pressure (RAP), pulmonary capillary wedge pressure (PCWP), lactate and cardiac power index (CPI).**

## Abbreviations

AH: hyperaemic areas
AO: occlusive areas
A-VBG: arterial and venous blood gas
BP: blood pressure
CI: cardiac index
CPI: cardiac power index
DO_2_: oxygen delivery
HR: heart rate
HSB: hypertonic sodium bicarbonate
HSL: hypertonic sodium lactate
LPS: lipopolysaccharide
MAP: mean arterial pressure
MPAP: mean pulmonary arterial pressure
NC: 0.9% NaCl
OE: oxygen extraction
OER: oxygen extraction ratio
PaO_2_/FiO_2_: arterial oxygen partial pressure and inspired oxygen fraction ratio
PBP: pulmonary blood pressure (PBP)
PCWP: pulmonary capillary wedge pressure, PF: peak flow
PU: perfusion units
Pv-aCO_2_: venoarterial CO2 tension difference
PVR: pulmonary vascular resistance
RAP: right atrial pressure
RF: rest flow
SID: strong ion difference
SvO_2_: mixed venous oxygen saturation
SVR: systemic vascular resistance
VO_2_: global oxygen consumption
